# Hematological and Biochemical Alterations Induced by Sub-Acute Administration of Permethrin in Rats

**DOI:** 10.3390/jox15060183

**Published:** 2025-11-01

**Authors:** Liliana Carmona-Aparicio, Elvia Coballase-Urrutia, Marisol Orozco-Ibarra, Norma Serrano-García, Silvia Caballero-Salazar, Maritza Ramírez-Pérez, Liliana Rivera-Espinosa, María E. Hernández, Hortencia Montesinos-Correa, Diana L. Pérez-Lozano, Daniel Diaz

**Affiliations:** 1 Laboratorio de Farmacología, Instituto Nacional de Pediatría, Ciudad de México 04530, Mexico; c_apariccio@yahoo.com.mx (L.C.-A.); lili_rives@yahoo.com (L.R.-E.); 2 Departamento de Bioquímica, Instituto Nacional de Cardiología Ignacio Chávez, Ciudad de México 14080, Mexico; marisol.ibarra@cardiologia.org.mx (M.O.-I.); maritza_ramirez@ciencias.unam.mx (M.R.-P.); 3 Departamento de Neurofisiología, Instituto Nacional de Neurología y Neurocirugía, Manuel Velasco Suárez, Ciudad de México 14269, Mexico; norma.serrano@innn.edu.mx; 4 Laboratorio de Oncología Experimental, Instituto Nacional de Pediatría, Ciudad de México 04530, Mexico; caballerosalazars@gmail.com; 5 Subdirección de Investigación Clínica, Instituto Nacional de Psiquiatría, Ciudad de México 14370, Mexico; drosoph2001@yahoo.com.mx; 6 Servicio de Endocrinología, Instituto Nacional de Pediatría, Ciudad de México 04530, Mexico; hortenciamontesinoscorrea@yahoo.com; 7 Licenciatura en Medicina General y Comunitaria, Universidad de la Salud, Campus Santa Fé, Ciudad de México 1345, Mexico; diana.perezl@unisa.cdmx.gob.mx; 8 Unidad de Inteligencia en Salud Pública, Centro de Investigación en Evaluación y Encuestas, Instituto Nacional de Salud Pública, Cuernavaca 62100, Morelos, Mexico; ddiaz@ciencias.unam.mx

**Keywords:** pyrethroids, hematology, synthetic pesticides, oxidative stress, enzymatic function

## Abstract

Permethrin (PERM) is a synthetic pyrethroid insecticide initially regarded as low risk. However, evidence now indicates that misuse and prolonged exposure can damage multiple physiological systems by disrupting enzymatic functions in subcellular structures. In this study, male Wistar rats were administered PERM (75, 150, or 300 mg/kg/day) for 15 days to assess its effect on hematological and biochemical parameters, including oxidative stress markers in the liver, kidney, and heart. Subacute PERM administration induced significant, dose-dependent toxicological alterations in exposed animals. Hematological analysis revealed impaired hematopoiesis, characterized by increased erythrocytes and platelets alongside decreased hemoglobin, hematocrit, mean corpuscular volume, and red cell distribution width. Biochemical analysis revealed elevated liver enzymes and bilirubin, along with reduced albumin levels, indicating hepatic alterations associated with PERM. The assessment of oxidative stress revealed tissue-specific responses following PERM exposure. While GPx, CAT, and SOD levels remained unchanged, GR activity increased in the heart, and GST activity increased in the liver. Additionally, a substantial decrease in MDA was observed in both the liver and heart. These collective alterations found in PERM-subacute exposed rats suggest the potential for cellular damage with the possible development of chronic pathologies, warranting further investigation.

## 1. Introduction

Pyrethroids belong to a broad-spectrum insecticide family and are synthetic derivatives of naturally occurring pyrethrins, which are extracted from the chrysanthemum flower *Chrysanthemum cinerariefolium*; since this plant was the first to be studied, it was observed that it could naturally exert an insecticidal effect. Studies on the secondary metabolites produced were initiated, and the cause of the said effect was considered to be pyrethroids. They were initially hailed as safe for farm animals and humans [1,2] and thus became the primary defense against household pests. Permethrin (PERM), a pyrethroid with a broad spectrum of activity, has been heavily used in both rural and urban areas, primarily due to its effectiveness in agriculture, controlling domestic pests, and in veterinary medicine [3,4]. Additionally, insecticides derived from plants (such as PERM) can serve as a basis for safe, effective, and environmentally sustainable bioformulations in pest control [5].

PERM is a chemical compound commonly used in several household products, including sprays, bombs, insect repellents, pet shampoos, lotions, and products focused on treating or eliminating skin parasites such as scabies and lice in humans [3]. PERM use has been met with concerns mainly due to its higher exposure to humans from the environment and metabolic concentration in human tissues. In vivo and in vitro studies have demonstrated that PERM affects the reproductive, skeletal, cardiovascular, immune, central, and peripheral nervous systems. Additionally, PERM acts as a cardiotoxicant, induces endocrine-disrupting activity, and is a hepatotoxin and cytotoxin, altering the respiratory chain [6,7]. Metabolites of pyrethroids, including 3-phenoxybenzoic acid (3-PBA), have been detected at high concentrations in human urine, plasma, brains, and livers [8] and have induced numerous biomolecules other than the parent PERM [9,10].

While there is sufficient data on the acute toxicity of PERM in humans, its subacute effects—such as changes in hematological and biochemical parameters, as well as oxidative stress markers in vital organ homogenates—have not been fully evaluated, creating a definitive requirement to assess these interactions to understand the real risks and pathologies it poses to human health. The demonstrated impact of PERM on multiple physiological systems, including the reproductive, skeletal, cardiovascular, immune, and nervous systems, alongside its clear hepatocarcinogenicity and endocrine-disrupting activity, underscores its potential danger. Consequently, there is a need for acute toxicity tests with subacute and chronic exposure assessments to provide a comprehensive toxicological evaluation. Research using animal models is crucial for elucidating the impact of repeated exposure on health at the biochemical, hematological, and functional levels. The present study was conducted to assess the subacute effects of PERM on hematological and biochemical parameters, including oxidative stress markers, in the liver, kidneys, and heart of Wistar rats, when administered 75, 150, or 300 mg/kg/day PERM for 15 days.

## 2. Materials and Methods

### 2.1. Reagents and Experimental Subjects

All reagents and chemicals were purchased from Sigma-Aldrich (St. Louis, MO, USA). Sodium pentobarbital was obtained from Pisabental^®^, PiSA Agropecuaria (Guadalajara, Jalisco, Mexico). All analytical reagents were purchased from J.T. Baker (Xalostoc, Estado de México, México). All other chemicals used in this study were of reagent grade and were commercially available.

Experimental Male Wistar rats weighing 150–180 g (5–6 weeks old) were used in this study. The rats were housed in a standard laboratory-controlled environment with a temperature of 21 ± 1 °C, relative humidity of 50–60%, and a 12 h light/dark cycle. Standard commercial chow (Harlan Teklad Global diet 2018S, sterilized; Harlan Teklad, Madison, WI, USA) and reverse osmosis-treated tap water were provided *ad libitum* to the rats. All procedures were conducted in accordance with the Official Mexican Norms regarding the use and care of laboratory animals (NOM-062-ZOO-1999) [11] and the disposal of biological residues (NOM-087-ECOL-1995) [12]. All procedures in the present study were performed in accordance with project 060/2018, following approval from the Institutional Committees for Research and Laboratory Animal Use and Care, and were also registered with COFEPRIS (17CI090031009).

**Figure 1:**
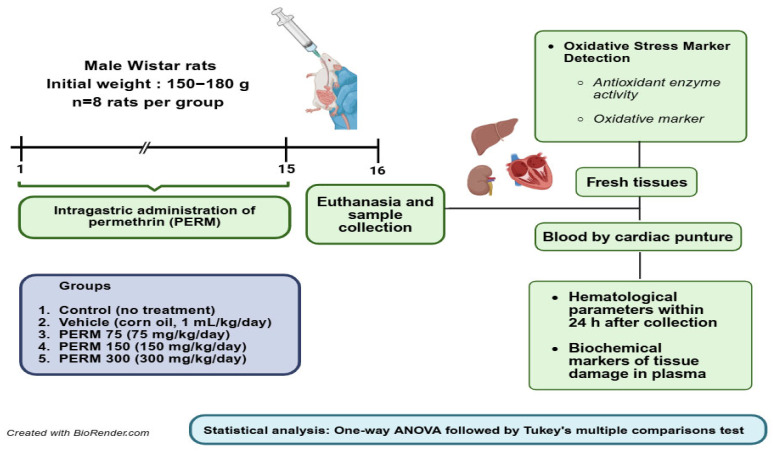
Experimental design and timeline of experimental procedures. Created in BioRender (own elaboration) https://BioRender.com/e9l06q6, accessed on 7 August 2025.

**Figure A1:**
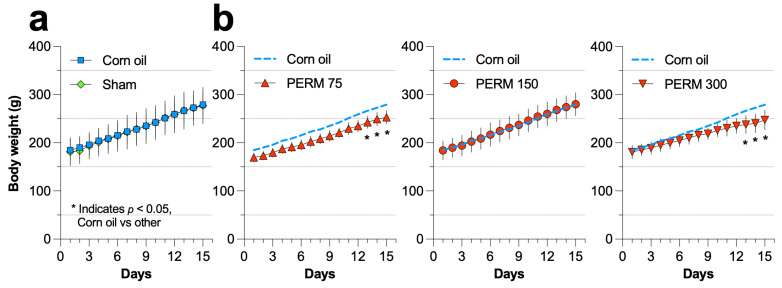
Effect of sham, corn oil, and PERM (75, 150, and 300 mg/kg) on the body weight of the rats included in each experimental group. (**a**) Comparison between the control group (corn oil) and the sham group, and (**b**) comparison between corn oil and PERM-treated rats. Results are expressed as mean ± SD (n = 8 per group). Statistical differences were presented on days 13–15 for PERM 75 (F = 10.28, *p* < 0.0001) and PERM 300 (F = 33.45, *p* < 0.0001).

Male rats were orally treated with PERM dissolved in corn oil via gastric intubation once daily for 15 days, with eight rats per group. Rats were not starved overnight before dosing. The animals were randomly assigned using a random number generator into one of five groups: Group 1, Sham (handled but no treatment); Group 2, Vehicle (corn oil, 1 mL/kg/day); Group 3, PERM 75 (75 mg/kg/day); Group 4, PERM 150 (150 mg/kg/day); and Group 5, PERM 300 (300 mg/kg/day). During the 15-day experimental period, the individual body weight of every rat within each group was recorded daily. As shown in Figure A1, except for PERM 75 and 300, which exhibited a significant difference with respect to the Corn oil group at the end of the period (days 13–15), no significant differences in the body weight of the rats were observed. Forty-eight hours after the last PERM dose, rats were euthanized with a lethal intraperitoneal injection of pentobarbital (0.6 mL/kg). Blood samples were collected via cardiac puncture, and fresh tissues from the liver, kidney, and heart were harvested for analysis (Figure 1).

### 2.2. Hematology Count and Quantification

Hematological parameters in whole blood (erythrocytes, hemoglobin, hematocrit, platelets, mean cell volume, erythrocyte distribution width, leukocytes, lymphocytes, neutrophils, monocytes, eosinophils, and basophils) were measured in triplicate using an autoanalyzer (Dimension AR, Dade Behring Inc., Newark, DE, USA). All analyses were performed within 24 h of blood sample collection.

### 2.3. Biochemical Markers of Tissue Damage in Serum

We identified several useful plasma markers that indicate potential damage to different organs resulting from exposure to varying concentrations of PERM. Blood was obtained by cardiac puncture and placed in a tube containing sodium citrate, which acts as an anticoagulant. The blood was then separated by centrifugation at 600× *g* for 15 min to obtain the serum. Alanine transaminase, aspartate aminotransferase, gamma-glutamyl transferase, albumin, total bilirubin, total protein, globulins, blood urea nitrogen, creatinine, alkaline phosphatase, C-reactive protein, glucose, triglycerides, and cholesterol were measured in triplicate using the adaptation of the methodology recommended by the International Federation of Clinical Chemistry, with an autoanalyzer (Dimension AR, Dade Behring Inc., Newark, DE, USA).

### 2.4. Tissue Homogenization and Total Protein Determination

After euthanasia, fresh tissue from the liver, kidneys, and heart was harvested and stored at −70 °C until analysis. For this, the tissue was homogenized in a 1:10 *w*/*v* ratio in cold phosphate buffer (50 mM, pH 7.0) containing 0.05% Triton X-100 using a Polytron PT2500C (Kinematica/Thermo Fisher Scientific Lucerne, Switzerland), with the homogenate maintained on ice. The homogenate was centrifuged at 10,000× *g* for 30 min in a refrigerated Centrifuge Micro 17R, Sorvall/ Thermo Scientific, Osterode am Harz Germany). The obtained supernatant was used to determine the activity of antioxidant enzymes and MDA content. The total protein concentration was determined through the Lowry method using an 8-point standard curve of bovine serum albumin (BSA).

### 2.5. Antioxidant Enzyme Activity and MDA Detection

To better understand the possible mechanism of PERM-induced damage, the activities of glutathione peroxidase (GPx), glutathione reductase (GR), glutathione S-transferase (GST), superoxide dismutase (SOD), and catalase (CAT), as well as their effects on lipid peroxidation, were evaluated by measuring malondialdehyde (MDA) levels in three vital organs, including the liver, kidney, and heart. CAT activity was assessed using the method previously described [13]. The results were expressed as units (μmoles of H_2_O_2_ decomposed per minute)/mg of protein. GPx activity was measured by the method of Lawrence and Burk [14], and the results were expressed as units per mg of protein. GR activity was assessed using the technique of Mannervik [15]. GST activity was evaluated using the Gronwald and Plaisance [16] method and the Habig method [17]. SOD activity was measured using the technique of Mori et al. [18] and Ukeda et al. [19]. The results were expressed as units per milligram of protein. MDA levels were measured by the method previously described [20]. All quantifications were performed in triplicate.

### 2.6. Statistical Analysis

The experimental groups were compared using a one-way analysis of variance (ANOVA). Before performing ANOVA, the normal distribution of the data was tested using the D’Agostino & Pearson omnibus normality test. The null hypothesis (H_0_) stated that values followed a normal distribution. Additionally, to test the homogeneity of the variances, Bartlett’s test was used. If the assumption that standard deviations were equal (H_0_) was satisfied, we employed ordinary one-way ANOVA and Brown–Forsythe ANOVA if heteroscedasticity was present. If the ANOVA null hypothesis (H_0_ = all group means were equal) was rejected (*p* < 0.05), Post hoc comparisons were performed using the Corn oil and Sham groups as the reference control groups, without comparing between PERM groups. For ordinary ANOVA, Dunnett’s multiple comparison test was applied using each reference group (corn oil or sham). For Brown-Forsythe ANOVA, Dunnett’s T3 multiple comparison test was used. For hematological and biochemical parameters, a linear trend analysis was conducted to determine if there was a dose-dependent effect of treatment on the response variable. Treatment was treated as sequential, assuming a left-to-right ordering (control → PERM 300), with the null hypothesis H_0_ = no linear trend. The test fits a linear regression model to estimate the slope in the response variable as the PERM dose increases. All the results are presented as the mean ± SD, and a *p* < 0.05 was deemed statistically significant. All tests and graphs were produced using Prism 10 (GraphPad Software, Inc., San Diego, CA, USA).

## 3. Results

### 3.1. Hematological Profile

**Figure 2:**
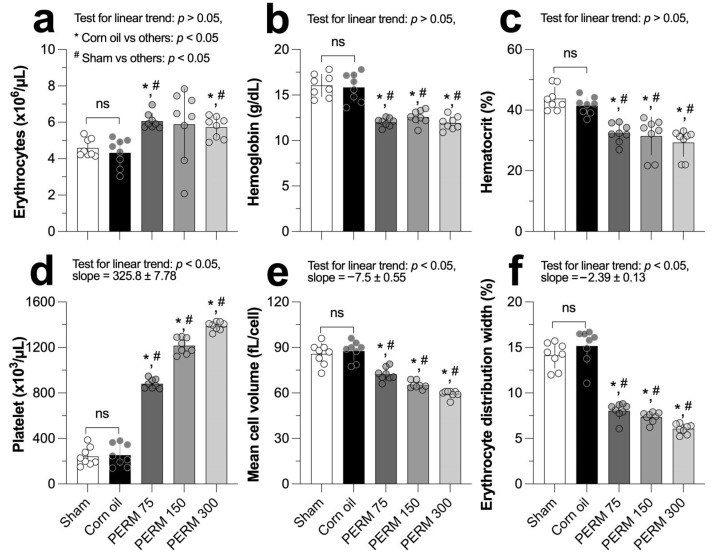
Effect of Sham (untreated), corn oil (vehicle), and PERM (75, 150, and 300 mg/kg) on (**a**) erythrocytes, (**b**) hemoglobin, (**c**) hematocrit, (**d**) platelets, (**e**) mean corpuscular volume, and (**f**) erythrocyte distribution width. Results are expressed as the mean ± SD. The experimental groups (n = 8 per group) were compared using one-way ordinary analysis of variance (ANOVA) followed by a multiple comparison test between each reference control group (corn oil and sham) and PERM-treated groups. A test for linear trend was also performed, assuming left-to-right group order. ANOVA results: erythrocytes, F (4, 12.5) = 4.86, *p =* 0.0136; hemoglobin, F (4, 22.1) = 33.45, *p* < 0.0001; hematocrit, F (4, 35) = 18.21, *p* < 0.0001; platelets, F (4, 35) = 469.3, *p* < 0.001; mean cell volume, F (4, 22.0) = 49.59, *p* < 0.0001; and erythrocyte distribution width, F (4, 18.9) = 98.29, *p* < 0.001. ns = *p* > 0.05.

As shown in Figure 2a, PERM 75 and 150 caused a significant increase in the erythrocyte count in rats (6.06 ± 0.43 and 5.88 ± 1.96 × 10^6^/μL) compared to the corn oil group (4.32 ± 0.79) and the sham group (4.59 ± 0.49). In contrast, the hemoglobin concentration decreased consistently in the experimental groups (range, 11.91 to 12.58 g/dL) in comparison to the control reference groups (corn oil, 15.85 ± 1.54 and sham, 16.06 ± 1.23; Figure 2b). Similarly, the hematocrit exhibited a significant decrease across the experimental groups, whose values ranged between 29.34% and 32.39%, and were lower than both reference control groups (corn oil, 41.51 ± 2.86 and sham, 43.82 ± 3.88, respectively; Figure 2c).

Platelet counts increased linearly (slope = 325.8 ± 7.78, *p* < 0.001) after PERM administration, passing from 881.6 ± 40.5 in PERM 75 to its final highest concentration at 1389.0 ± 37.1 × 10^3^/µL in the PERM 300 group, whose values were significantly above both the corn oil group (253.0 ± 97.7) and the sham group (241.4 ± 81.7, Figure 2d). On the contrary, mean cell volume and erythrocyte distribution width (Figure 2e,f) both exhibited a downward trend as the PERM concentration increased, with a slope estimated at −7.5 ± 0.55 for mean cell volume and −2.3 ± 0.13 for erythrocyte distribution width. Notably, across all hematological markers, there were no significant differences between the corn oil and the sham control reference groups (*p* > 0.05). All the estimations are summarized in Table A1.

### 3.2. White Blood Cell Count

**Figure 3:**
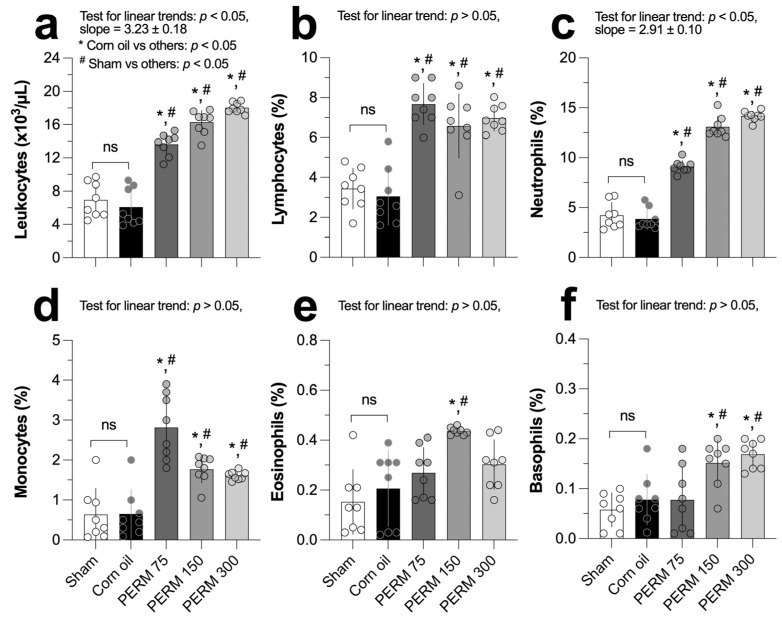
Effect of Sham (untreated), corn oil (vehicle), and PERM (75, 150, and 300 mg/kg) on (**a**) leukocytes, (**b**) lymphocytes, (**c**) neutrophils, (**d**) monocytes, (**e**) eosinophils, and (**f**) basophils. Results are expressed as the mean ± SD. The experimental groups (n = 8 per group) were compared using one-way ordinary analysis of variance (ANOVA) followed by a multiple comparison test between each reference control group (corn oil and sham) and PERM-treated groups. A test for linear trend was also performed, assuming left-to-right group order. ANOVA results: leucocytes, F (4, 25.2) = 85.30, *p <* 0.0001; lymphocytes, F (4, 35) = 25.41, *p* < 0.0001; neutrophils, F (4, 35) = 201.7, *p* < 0.0001; monocytes, F (4, 22.7) = 20.34, *p* < 0.0001; eosinophils, F (4, 24.8) = 7.65, *p =* 0.004; and basophils, F (4, 35) = 9.16, *p* < 0.0001. ns = *p* > 0.05.

Compared to the corn oil and sham control reference groups, leukocytes increased significantly and gradually across the PERM-treated groups, with a slope of 3.23 ± 0.18 (*p* < 0.0001; Figure 3a). Following PERM administration, the percentage of lymphocytes increased significantly compared to both reference groups (3.05 ± 1.43 and 3.44 ± 1.01 for corn oil and sham groups, respectively) but with comparable values among the treated groups (range, 6.5% to 7.6%; Figure 3b). In comparison to the corn oil group (3.86 ± 1.05) and the sham group (4.21 ± 1.31), the percentage of neutrophils increased significantly and linearly with a slope of 2.91 ± 0.10 (*p* < 0.001, Figure 3c) among the experimental groups (PERM 75, 9.11 ± 0.62; PERM 150, 13.09 ± 1.06; PERM 300, 14.18 ± 0.49%).

After exposing the rats to PERM, the percentage of monocytes in their blood increased significantly compared to the reference groups, with PERM 75 being the group that exhibited the highest value (2.81 ± 0.82%, Figure 3d). As depicted in Figure 3e, the percentage of eosinophils was significantly higher in the PERM 150 group (0.43 ± 0.01%) compared to the corn oil group (0.20 ± 0.15%) and the sham group (0.15 ± 0.12%). Finally, exposing the rats to the two highest concentrations of PERM resulted in a significant increase in the percentage of basophils (PERM 150, 0.15 ± 0.04 and PERM 300, 0.16 ± 0.02) when compared to the reference groups (corn oil, 0.07 ± 0.05 and sham, 0.05 ± 0.03; Figure 3f). Across the parameters, the rats from the corn oil and the sham groups exhibited similar values (*p* > 0.05). All the estimations are summarized in Table A1.

### 3.3. Biochemical Profile

**Figure 4:**
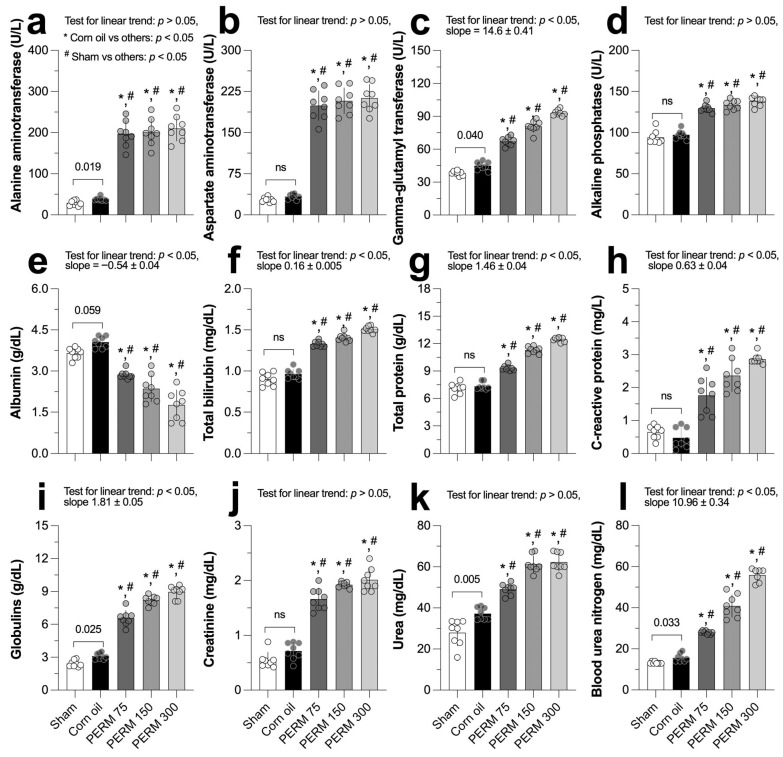
Effect of Sham (untreated), corn oil (vehicle), and PERM (75, 150, and 300 mg/kg) on bi-ochemical parameters. Results are expressed as mean ± SD. Experimental groups (n = 8 per group) were compared using one-way ordinary analysis of variance (ANOVA) followed by a multiple comparison test between each reference control group (corn oil and sham) and PERM-treated groups. A test for linear trend was also performed, assuming left-to-right group order. ANOVA results: (**a**) alanine aminotransferase, F (4, 21.7) = 113.3, *p* < 0.0001; (**b**) aspartate aminotransferase, F (4, 21.6) = 192.3, *p* < 0.0001; (**c**) gamma-glutamyl transferase, F (4, 35) = 314.5, *p* < 0.0001; (**d**) alkaline phosphatase, F (4, 35) = 99.7, *p* < 0.0001; (**e**) albumin, F (4, 19.2) = 53.43, *p* < 0.0001; (**f**) total bilirubin, F (4, 35) = 212.7, *p* < 0.0001; (**g**) total protein, F (4, 35) = 293.6, *p* < 0.0001; (**h**) C-reactive protein, F (4, 21.4) = 62.2, *p* < 0.001; (**i**) globulins, F (4, 35) = 314.5, *p* < 0.0001; (**j**) creatinine, F (4, 26.6) = 145.1, *p* < 0.001; (**k**) urea, F (4, 35) = 96.5, *p* < 0.0001; (**l**) blood urea nitrogen, F (4, 13.7) = 268.0, *p* < 0.001 ns = *p* > 0.05.

All the estimations of the PERM effect on the biochemical markers are summarized in Table A2. Following subacute PERM administration, alanine aminotransferase (Figure 4a), aspartate aminotransferase (Figure 4b), and alkaline phosphatase (Figure 4d) levels increased in all three experimental groups compared to both control reference groups. Gamma-glutamyl transferase showed a linear increase (slope = 14.6 ± 0.41) as the PERM concentration augmented, passing from 38.25 ± 1.98 and 44.88 ± 3.79 U/L in the sham and the corn oil groups, respectively, to 93.25 ± 2.65 in the PERM 300 group (Figure 4c).

Albumin levels showed a significant and linear reduction with an estimated slope of −0.54 ± 0.04 after exposing the rats to increasing PERM concentrations (Figure 4e). In contrast, total bilirubin, total protein, and C-reactive protein levels all tend to increase linearly, though with a variable slope (range, 0.16 to 1.46) in the PERM-exposed rats (Figure 4f–h). Similarly, PERM treatment induced a linear increase in both globulins and blood urea nitrogen in the exposed rats, causing globulins to increase from 3.1 ± 0.27 and 2.42 ± 0.29 g/dL in the corn oil and sham groups, respectively, to their highest levels at PERM 300 (8.96 ± 0.56 g/dL; Figure 4i). Blood urea nitrogen changed from 15.63 ± 1.92 and 13.25 ± 0.46 in the corn oil and sham groups, respectively, to 55.86 ± 3.23 g/dL at PERM 300 (Figure 4l). The other two renal function indicators, creatinine and urea, also showed nephrotoxic effects associated with PERM exposure because their levels increased significantly above the reference groups (Figure 4j and 4k, respectively).

In response to PERM exposure, the glucose concentration in experimental rats increased significantly compared to the control reference group (76.75 ± 8.89 and 73.63 ± 7.11 mg/dL for corn oil and sham, respectively; F (4, 35) = 276.5, *p* < 0.001). Additionally, glucose levels increased linearly as the PERM concentration increased (slope = 22.41 ± 0.69, *p* < 0.0001): PERM 75, 120.6 ± 5.06 mg/dL; PERM 150, 135.9 ± 2.85 mg/dL; and PERM 300, 156.1 ± 5.33 mg/dL. Regarding triglycerides and cholesterol, both increased significantly in the PERM-treated groups compared to the control reference groups. Triglycerides increased significantly from 80.0 ± 6.95 and 72.5 ± 16.29 mg/dL in the corn oil and the sham groups, respectively, to a range between 127.6 and 138.9 mg/dL across the PERM-treated rats (F (4, 16.8) = 94.27, *p* < 0.0001), though with no linear trend. Finally, cholesterol levels remained unchanged in the PERM-treated rats (60.0 ± 3.7, 59.3 ± 5.2, and 66.1 ± 3.5 mg/dL for PERM 75, 150, and 300, respectively) when compared to their control counterparts (corn oil, 61.5 ± 5.50 and sham, 57.3 ± 2.26; F (4, 35) = 4.84, *p* > 0.05). In all three biochemical parameters, there were no significant differences between the sham and corn oil groups (*p* > 0.05) (Table A2).

Despite half of the biochemical parameters assessed in the study showing significant differences between the corn oil and sham groups (*p* < 0.05), the PERM groups exhibited the same statistical differences as the control reference groups.

### 3.4. Antioxidant System in Liver, Kidney, and Heart

The effects of subacute PERM exposure on the antioxidant enzyme activity of the liver, kidney, and heart of the treated rats are summarized in Table 1. GPx, CAT, and SOD activity remained unchanged in all three examined tissues of PERM-treated rats compared to the corn oil and sham groups. In contrast, GR activity in the heart was significantly reduced in the PERM 300 group (0.003 ± 0.001 U/mg) compared to the reference groups (0.006 ± 0.001 and 0.008 ± 0.001 U/mg for corn oil and sham, respectively), with no differences observed in the other two tissues. In the liver, PERM administration at 150 and 500 mg/kg increased GST activity by 0.095 and 0.103 U/mg, respectively, compared to the corn oil group (0.062 ± 0.02) and the sham group (0.063 ± 0.007). No further effect was observed in the kidney and heart. Finally, MDA levels decreased significantly in the liver in the PERM 150 and 300 groups. Meanwhile, MDA levels in the heart were reduced at 300 mg/kg PERM (0.59 ± 0.2 mmol/mg) compared to the values of 1.10 × 10^6^ ± 0.27 and 96 × 10^6^ ± 0.24 mmol/mg observed in the rats from the corn oil and sham groups, respectively.

## 4. Discussion

Pesticides are widely used on farms and in homes, posing a significant risk to both humans and animals. Furthermore, it is present in various formulations for common therapeutic and topical use, such as pediculicidal treatments and dermatological preparations, which are even used in the pediatric population. They can be harmful directly or through the food they eat. Pyrethroids, such as PERM, are generally considered safe for use. However, as their use becomes more prevalent, it has been observed that they can induce health problems. Therefore, research focuses on how we affect the environment and its functioning. To maintain the health of animals and ensure proper nutrition and treatment, their physical weight must be considered to observe how different doses of PERM affect them. We observed that the weight of animals exposed to different PERM treatments was not altered, unlike what has been reported by other authors, who have noted metabolic alterations associated with insulin resistance, direct damage to the liver and kidneys, and increased body weight. It is worth mentioning that these studies were conducted in females, and that the exposure period was up to 12 weeks, differences that may account for the different results reported (Appendix A) [21,22]. It is worth mentioning that in our experimental groups, we only had one death in the 300 mg/kg regimen (4%), which was replaced to complete the n. However, there are reports of mortality in PERM treatments in murine models, which are described in doses greater than 400 mg/kg.

Hematologic parameters and biochemical markers indicate the proper health and function of major organs, such as the heart, kidney, and liver, as well as systemic metabolic functions. Therefore, alterations of these parameters are usually precursors of damage to the organs or functional deterioration of the organism. Nevertheless, relatively little is known about the hematological and biochemical issues induced by subacute oral exposure to PERM. Our data demonstrated an increase in erythrocytes and platelets, with significant reductions in hemoglobin, hematocrit, mean cell volume, and erythrocyte distribution width. The finding that erythrocyte values increased in the PERM-treated groups is in agreement with previous findings [23]. Still, it contrasts with other studies that did not find differences between the treated groups and the control [24,25]. Our results showed that hemoglobin levels decreased, thus contrasting with previous findings that reported an increase in this parameter [24]. Similar to our result, Shearer et al. [26] reported that in a population exposed to PERM, both red blood cell counts and hemoglobin levels decreased. We observed a significant increase in hematocrit values, while erythrocyte distribution width decreased in a dose–response trend. Likewise, mean cell volume C values decreased linearly as the PERM concentration increased. Taiwo Idowu et al. [23] reported that they did not observe significant differences in the previous variables. Another study reported that hematological values at 15 days of treatment showed a decrease, but hemoglobin values remained unchanged after PERM exposure [27]. Similar results have been observed in field workers exposed occupationally, suggesting that the reduction in hemoglobin can be attributed to the decreased size of red blood cells or to the impairment of heme biosynthesis in the bone marrow [28,29,30]. Our study, as well as other research, suggests that PERM exposure may induce hematopoietic alterations. This could be due to impaired heme synthesis in the bone marrow and a decreased red blood cell count, as demonstrated in other studies [28,29]. Furthermore, the treated groups showed an increase in hematocrit (Hct), with a similar effect on red blood cells. This confirms that PERM induces changes in the hematopoietic system. We also observed a decrease in mean cell volume in PERM-treated groups, which could indicate anemia characterized by abnormalities in erythrocyte size.

The effects of PERM could be attributed to the high reactivity of its molecules, due to the presence of ketone and aromatic benzene groups in its chemical structure. This structure facilitates electron sharing and can form complexes with iron [31]. This interaction leads to a loss of functional iron from intracellular sites, resulting in iron deficiency and disruption of iron homeostasis. These effects contribute to altered hematocrit and mean cell volume. Notably, Honda et al. [30] [32] reported that low iron levels can induce anemia; these alterations could be due to changes in blood production. From a biological perspective, PERM can modify oxygen transport dynamics, impact blood cell production, and ultimately alter physiological homeostasis [23]. Likewise, in our model, platelets had a dose-dependent response. In agreement with Al-Damegh [33], who reported that administration of synthetic type I pyrethroids induces an elevation of total corpuscular volume of red blood cells and platelets at different exposure times in rats (24, 48, and 72 h), supporting our findings.

One study examined blood cell counts in insecticide plant workers, some of whom were exposed to PERM. Researchers collected blood samples from 99 workers who were exposed to insecticides and 107 workers who were not. The insecticide-exposed group had significantly more cases of high platelet counts. Furthermore, their white blood cell counts, platelet counts, and red blood cell counts were all higher, suggesting that exposure to insecticides alters their blood composition [34]. In our experimental model, PERM administration significantly altered blood counts, including increased counts of white blood cells, lymphocytes, leukocytes, neutrophils, monocytes, eosinophils, and basophils. Previous research supports our findings [25]. However, other investigations reported no changes in lymphoid cells after exposure to a type I pyrethroid [26,35]. On the other hand, an increase in white blood cells, especially lymphocytes and monocytes, can generally indicate that the immune system is responding to fight an infection, injury [1], or inflammation [36], which can damage tissues and organs.

At the biochemical level, a dose-dependent increase in liver enzymes (alanine aminotransferase, aspartate aminotransferase, gamma-glutamyl transferase, alkaline phosphatase, and total bilirubin) was observed, which are clear markers of liver damage. Similar results were demonstrated by other studies [21,37]. On the other hand, albumin decreased significantly, as previously reported after long-term exposures [38]. Since the liver primarily synthesizes albumin, direct hepatocellular damage would likely alter protein synthesis. These alterations may reflect a loss of functional integrity of the hepatocyte membrane, revealing that PERM not only affects the liver at a functional level but also induces considerable cellular stress that can trigger organ dysfunction [37,38]. Local tissue damage is known to trigger an inflammatory reaction, which can intensify an acute phase response. This can lead to an increase in several plasmatic proteins, such as total protein and C-reactive protein, which in turn lead to an increase in immunoglobulins [39]. We observed an increase in total protein, C-reactive protein, and globulins. Kawther et al. [40] observed a slight increase in C-reactive protein in patients treated with 5% PERM, and there are no reports on animal models. Likewise, creatinine, urea, and urea nitrogen levels increased dose-dependently; similar observations have been reported in several studies [21,41,42]. These markers can measure kidney filtration efficiency and evaluate protein degradation. It has been proposed that PERM may accelerate the aging process by decreasing the glomerular filtration rate and affecting sodium excretion [43]. In our study, cholesterol showed significant differences only at the highest concentration, and urea increased in a dose-dependent manner. We also reported significant differences in glucose, triglycerides, and cholesterol; similar results were reported by [22,32].

Regarding the determination of antioxidant enzyme activity, we found that GPx, CAT, and SOD were not significantly modified in the organs of interest (liver, kidney, and heart). However, GR activity decreased in the heart, and GST activity was higher in the liver. Unexpectedly, MDA (which indicates the extent of lipid peroxidation) was significantly reduced in the hearts and kidneys exposed to the highest PERM doses. The above results do not indicate oxidative stress, but rather suggest changes in the availability of reactive oxygen species (ROS), which have been documented as key redox signaling molecules with beneficial basal levels that play a role in cellular signaling [43,44]. Therefore, we believe that the decrease in ROS levels could promote molecular processes adverse to cells. The above would need to be explored in depth in future research, considering the complexity of the antioxidant system.

The results of the present study provide evidences that chronic PERM exposure (75, 150 and 300 mg/kg/day, orally) in male rats could cause significant shifts in various physiological systems eliciting hematological and biochemical symptoms from changes in blood cell production to iron metabolism disturbances as well as liver function impairment indication by increases in total bilirubin and some hepatic damage markers with decreased albumin serum levels. In addition, selective alterations were found in the antioxidant enzymes (glutathione reductase in the heart and glutathione-S-transferase in the liver and heart), indicating an adaptation to oxidative stress. These findings are consistent with established modes of PERM toxicity, including mitochondrial dysfunction, the overproduction of reactive oxygen species (ROS), and alterations to the cell membrane. It is also important to note that low-dose exposure, as observed in previous studies, induces ROS production and disrupts the balance of the antioxidant enzymatic system, while increasing the gene expression of proinflammatory interleukins in specific brain regions. These effects could subsequently lead to cellular damage due to mitochondrial dysfunction. An intriguing result was the decline in MDA, which could be misinterpreted as a reduction in oxidative stress. Previous work, however, indicates that preconditioning depends on the upregulation of antioxidant defenses in response to chronic exposures [44]. This is not a true enhancement, however, but rather compensation for cellular-level damage that can obscure deeper tissue injury. Thus, the findings in this study present novel evidence on the early processes leading to adaptation and injury following PERM. While broadening toxicological understanding, they underscore the importance of reinforcing the regulation of its use, establishing preventive public health measures, and conducting further studies on minimum doses and timing for these effects.

## 5. Conclusions

Our results showed that significant effects on blood parameters, iron metabolism, and antioxidant responses were observed in this study after 15 days of exposure to 75, 150, and 300 mg/kg/day of permethrin (PERM) in Wistar rats. Reduced albumin levels, increased total bilirubin, elevated liver injury markers, and changed blood cell production were all signs of impaired liver function following exposure. Antioxidant enzymes, such as glutathione reductase in the heart and glutathione-S-transferase in the liver and heart, also showed selective alterations. Although it does not entirely stop cellular damage, the observed decrease in MDA suggests an adaptive response that shields cells from oxidative stress to some extent. Exposure to PERM affects several physiological systems and may trigger compensatory processes that mitigate some of its harmful effects. More significantly, the findings highlight the importance of controlling PERM exposure for public health, even beyond the biochemical changes. To mitigate potential long-term risks to vital organs, stricter safety regulations and preventive measures are crucial. To better understand the effects of chronic exposure, improve risk assessments, and strengthen regulatory frameworks to safeguard human health and the environment, these results also underscore the need for additional toxicological research.

## Figures and Tables

**Table 1:** Effect of permethrin (PERM 75, 150, and 300 mg/kg body weight/day) on the activity of antioxidant enzymes in the liver, kidney, and heart of male rats treated for 15 days.

Parameter	Sham	Corn Oil	PERM			ANOVA Results
			75	150	300	
Liver						
GPX (U/mg)	0.153 ± 0.019	0.156 ± 0.028	0.148 ± 0.038	0.164 ± 0.047	0.177 ± 0.017	F (4, 25) = 0.73; *p* = 0.577
GR (U/mg)	0.007 ± 0.002	0.008 ± 0.005	0.007 ± 0.001	0.009 ± 0.001	0.012 ± 0.007	F (4, 11.4) = 1.21; *p* = 0.357
GST (U/mg)	0.063 ± 0.007 ^ns^	0.062 ± 0.020	0.068 ± 0.011	0.095 ± 0.016 *^,#^	0.103 ± 0.010 *^,#^	F (4, 25) = 11.41; *p* < 0.0001
CAT (KU/mg)	5415 ± 717	5433 ± 1332	5226 ± 1074	5583 ± 1744	4344 ± 1035	F (4, 25) = 0.97; *p* = 0.439
SOD (U/mg)	118 ± 6.8	112 ± 7.8	115 ± 7.9	125 ± 9.0	118 ± 5.7	F (4, 25) = 1.28; *p* = 0.302
MDA (mmol/mg)	72 × 10^6^ ± 15 × 10^6 ns^	69 × 10^6^ ± 16 × 10^6^	56 × 10^6^ ± 0.13 × 10^6^	46 × 10^6^ ± 13 × 10^6^ *^,#^	35 × 10^6^ ± 11 × 10^6^ *^,#^	F (4, 25) = 6.99; *p* = 0.0006
Kidney						
GPX (U/mg)	0.073 ± 0.011	0.069 ± 0.026	0.068 ± 0.018	0.092 ± 0.010	0.075 ± 0.011	F (4, 25) = 2.00; *p* = 0.125
GR (U/mg)	0.012 ± 0.002	0.011 ± 0.001	0.011 ± 0.004	0.016 ± 0.002	0.014 ± 0.004	F (4, 16.8) = 2.94; *p* = 0.051
GST (U/mg)	0.017 ± 0.003	0.015 ± 0.005	0.015 ± 0.005	0.019 ± 0.003	0.018 ± 0.002	F (4, 25) = 1.17; *p* = 0.346
CAT (KU/mg)	3114 ± 563	2895 ± 841	2613 ± 780	3577 ± 613	2781 ± 270	F (4, 25) = 1.99; *p* = 0.125
SOD (U/mg)	500 ± 23.7	355 ± 18.6	328 ± 19.9	321 ± 6.7	318 ± 6.6	F (4, 25) = 1.28; *p* = 0.302
MDA (mmol/mg)	107 × 10^6^ ± 50 × 10^6^	100 × 10^6^ ± 0.43 × 10^6^	109 × 10^6^ ± 0.23 × 10^6^	68 × 10^6^ ± 24 × 10^6^	90 × 10^6^ ± 27 × 10^6^	F (4, 25) = 1.33; *p* = 0.284
Heart						
GPX (U/mg)	0.014 ± 0.003	0.011 ± 0.003	0.011 ± 0.003	0.010 ± 0.005	0.0091 ± 0.002	F (4, 25) = 1.77; *p* = 0.166
GR (U/mg)	0.008 ± 0.001 ^ns^	0.006 ± 0.001	0.006 ± 0.002	0.005 ± 0.002	0.003 ± 0.001 *^,#^	F (4, 25) = 4.37; *p* = 0.008
GST (U/mg)	0.006 ± 0.008	0.008 ± 0.001	0.008 ± 0.001	0.007 ± 0.001	0.007 ± 0.001	F (4, 25) = 1.99; *p* = 0.126
CAT (KU/mg)	198.9 ± 78.1	229.8 ± 90.7	256.2 ± 61.0	229.7 ± 42.5	175.8 ± 55.6	F (4, 25) = 1.26; *p* = 0.309
SOD (U/mg)	1939 ± 78.8	1837 ± 67.8	1736 ± 57.4	2731 ± 80.31	2230 ± 7.20	F (4, 25) = 2.87; *p* = 0.100
MDA (mmol/mg)	96 × 10^6^ ± 24 × 10^6 ns^	110 × 10^6^ ± 27 × 10^6^	106 × 10^6^ ± 21 × 10^6^	77 × 10^6^ ± 26 × 10^6^	59 × 10^6^ ± 20 × 10^6^ *^,#^	F (4, 25) = 4.75; *p* = 0.005

Data are presented as mean ± SD (n = 6 per group). GPx = Glutathione peroxidase; GR = glutathione reductase; GST = glutathione-S-transferase; CAT = catalase; SOD = superoxide dismutase; MDA = malondialdehyde; ns = *p* > 0.05; * corn oil vs. others = *p* < 0.05; ^#^ sham vs. others = *p* < 0.05. Experimental groups were compared using one-way ordinary analysis of variance (ANOVA) followed by a multiple comparison test between each reference control group (corn oil and sham) and PERM-treated groups.

## Data Availability

The original contributions presented in this study are included in the article. Further inquiries can be directed to the corresponding author.

## Appendix A

**Table A1:** Effect of PERM treatment on the hematological parameters.

Parameter	Sham	Corn Oil	PERM		
			75	150	300
Erytrocyte (×10^6^/µL)	4.59 ± 0.49	4.32 ± 0.79	6.06 ± 0.43	5.88 ± 1.96	5.72 ± 0.59
Hemoglobin (g/dL)	16.06 ± 1.23	15.85 ± 1.54	12.01 ± 0.58	12.58 ± 0.76	11.91 ± 0.72
Hematocrit (%)	43.82 ± 3.88	41.51 ± 2.86	32.39 ± 3.09	31.51 ± 6.21	29.34 ± 4.59
Platelets (×10^3^/µL)	241 ± 81	253 ± 97	881 ± 40	1216 ± 70	1389 ± 37
Mean cell volume (fL/cell)	85.75 ± 7.21	87.70 ± 6.53	72.38 ± 4.16	65.28 ± 2.51	59.46 ± 2.41
Erythrocyte distribution width (%)	14.14 ± 1.42	15.17 ± 1.92	8.04 ± 0.87	7.35 ± 0.55	6.06 ± 0.53
Leucocytes (×10^3^/µL)	6.95 ± 2.07	6.08 ± 2.25	13.63 ± 1.44	16.28 ± 1.44	18.03 ± 0.64
Lymphocytes (%)	3.44 ± 1.02	3.05 ± 1.43	7.67 ± 1.03	6.57 ± 1.61	6.97 ± 0.65
Neutrophils (%)	4.21 ± 1.31	3.86 ± 1.05	9.11 ± 0.62	13.09 ± 1.06	14.18 ± 0.49
Monocytes (%)	0.63 ± 0.65	0.65 ± 0.61	2.81 ± 0.82	1.76 ± 0.33	1.60 ± 0.11
Eosinophils (%)	0.15 ± 0.12	0.21 ± 0.15	0.26 ± 0.10	0.43 ± 0.01	0.30 ± 0.1
Basophils (%)	0.06 ± 0.03	0.07 ± 0.05	0.07 ± 0.06	0.15 ± 0.04	0.16 ± 0.02

**Table A2:** Effect of PERM treatment on the biochemical parameters.

Parameter	Sham	Corn Oil	PERM		
			75	150	300
Erytrocyte (×10^6^/µL)	4.59 ± 0.49	4.32 ± 0.79	6.06 ± 0.43	5.88 ± 1.96	5.72 ± 0.59
Alanine aminotransferase (U/L)	28.73 ± 6.28	37.94 ± 4.55	196.50 ± 31.61	201.3 ± 32.60	209.9 ± 30.34
Aspartate aminotransferase (U/L)	27.50 ± 4.21	33.38 ± 4.59	199.4 ± 26.83	208.0 ± 23.71	212.90 ± 25.0
Gamma-glutamyl transferase (U/L)	38.25 ± 1.98	44.88 ± 3.79	67.25 ± 3.88	80.88 ± 5.35	93.25 ± 2.65
Alkaline phosphatase (U/L)	94.13 ± 7.90	97.83 ± 5.34	129.75 ± 4.74	133.62 ± 5.80	138.0 ± 5.55
Albumin (g/dL)	3.65 ± 0.20	4.05 ± 0.20	2.86 ± 0.15	2.36 ± 0.48	1.76 ± 0.55
Total bilirubin (mg/dL)	0.90 ± 0.08	0.96 ± 0.06	1.33 ± 0.03	1.40 ± 0.04	1.51 ± 0.03
Total protein (g/dL)	7.11 ± 0.58	7.35 ± 0.40	9.32 ± 0.27	11.31 ± 0.35	12.46 ± 0.22
C-reactive protein (mg/L)	0.65 ± 0.20	0.47 ± 0.31	1.76 ± 0.55	2.36 ± 0.49	2.86 ± 0.15
Globulins (g/dL)	2.42 ± 0.29	3.10 ± 0.27	6.56 ± 0.69	8.20 ± 0.40	8.96 ± 0.56
Creatinine (mg/dL)	0.54 ± 0.14	0.71 ± 0.15	1.66 ± 0.19	1.92 ± 0.05	2.01 ± 0.21
Urea (mg/dL)	27.94 ± 6.33	37.19 ± 3.02	49.13 ± 2.70	61.28 ± 4.12	62.30 ± 4.44
Blood urea nitrogen (mg/dL)	13.25 ± 0.46	15.63 ± 1.92	28.00 ± 0.75	40.88 ± 5.43	55.86 ± 3.23
Glucose (mg/dL)	73.63 ± 7.11	76.75 ± 8.89	120.62 ± 5.06	135.87 ± 2.85	156.12 ± 5.33
Triglyceride (mg/dL)	72.51 ± 16.29	80.03 ± 6.95	127.60 ± 4.88	135.20 ± 7.52	138.88 ± 6.23
Cholesterol (mg/dL)	57.38 ± 2.26	61.50 ± 5.50	60.0 ± 3.70	59.38 ± 5.23	66.13 ± 3.56
